# Instagram as an Educational Opportunity for Neurology and Neuroscience

**DOI:** 10.1212/NE9.0000000000200152

**Published:** 2024-11-08

**Authors:** Stefano Sandrone

**Affiliations:** From the Department of Brain Sciences, Faculty of Medicine, Imperial College London, United Kingdom.

## Background

Instagram is one of the most used social networks globally, with 2 billion active users per month.^1^ Thirty-two percent of global Instagram audiences are aged between 18 and 24 years, 30.6% between 25 and 34 years, and 16% between 35 and 44 years.^2^ Not only is there an overlap between the age of Instagram users and the average age of medical students, but patients increasingly use social media for information on their health and as a place to find support.^3^ Considering it was launched in October 2010, this app does not seem to receive the attention it deserves from the world of neurology and neuroscience education. In this article, we discuss ideas, limitations and opportunities for future educational applications of Instagram in neurology and neuroscience, and in the broader context of medical education research.

## Learners' Use of Instagram

Instagram's visual nature distinguishes it from text-focused social media.^4^ This makes it a valuable platform for sharing content, especially “when the aim is to display graphic images supplemented by educational content.”^5^ Most simply, learners can use Instagram to explore or refresh neuro-related content at their own pace. Instagram can host neurologic content through pictures (i.e., posts), videos (i.e., reels and stories) and accompanying text. Using hashtags renders the content more easily searchable and increases visibility. Case samples, special cases, short summaries of diseases or illustrations that facilitate learning about anatomical structures can take the form of a post or a reel.^6^

From an andragogical perspective, using Instagram for educational purposes aligns well with self-directed learning^7^ and the rising trends of microlearning and nanolearning. These two converge in many ways and are somewhat complementary: while the former gives the learner independence on the strategy and the pace of learning, the latter aims at improving or refreshing skills and competencies, thanks to its bite-sized, digestible format (often by push media) and its reduced “cognitive load” on the learners, be they students, patients, or caregivers.^8^

Only a few examples of lectures compressed and “pushed” through Instagram exist. Yet, exciting insights come from an infectious diseases pharmacotherapy course for pharmacy students where Instagram content corresponding to lecture objectives was developed and published for 12 weeks without academic or monetary incentives for learners.^5^ Of 234 respondents, 115 had followed the study content; 75% recognized that the experiment had enhanced their learning and 41% stated that they would encourage faculty to use Instagram for educational purposes (whereas it was only 19% before the study).^5^

Future works should first explore whether there is an actual desire to use Instagram in a specific cohort on a particular topic, both on the learners' and academic staff's side, and then assess the effect of short-term and long-term knowledge retention. Besides this, it would be beneficial to interview learners and staff who have used Instagram educationally and decided it was not for them^4^ to explore the reasons behind this. Testing how Instagram can work as a communication tool within learners' groups^9^ might be another avenue to follow because this app also allows sending private messages and instantly sharing content.

## Educators' Use of Instagram

### Recruitment and Prestige

The pandemic and physical distancing have accelerated the adoption of social media. After the coronavirus disease outbreak, neurology residency programs increased their social media presence. Instagram (along with Twitter) became home to the highest number of residency program accounts during the pandemic, a spot formerly held by Facebook (both Instagram and Facebook are owned by Meta Platforms, Inc.): 73.8% of residency Instagram accounts were created after March 1, 2020.^10^ A correlation has even been found between the presence and number of social media accounts and program prestige (linked to research output, funding and number of residents pursuing a fellowship and ranked with Doximity). A strong correlation exists between the program quartile and the presence of 1 or more social media accounts (*R*^2^ = 0.93) and between the program quartile and the number of social media accounts (*R*^2^ = 0.97).^10^

Researchers could check whether there is a correlation between the global ranking and the number of followers, likes and comments at an institutional level, and spot eventual exceptions to the ranking, which can be further investigated. At an individual level, it can be calculated to what extent the academic productivity of neurologists is positively or negatively correlated with the number of followers (and, perhaps, even with the time spent on Instagram, which is recorded by the app).

### Journals, Societies and Foundations

Virtually all neurologic and neuroscience organizations, journals and foundations have an Instagram account.^11^ Having a social media presence has been hailed as a necessity since the launch of Instagram. Accounts of journals and institutions share neurologic cases^6^ and quizzes with questions such as “Where is the lesion?” or “What is the diagnosis?”. Instagram immediately highlights the correct answer, which is an advantage as this provides immediate feedback. From the perspective of an educator or a content creator, using a game-based approach^12^ makes learning even more rewarding^13^ and taps into user engagement.

On this note, Instagram can be used for public engagement activities. However, few studies have quantified the impact of Instagram as a public engagement tool in neurology.^14^ Instagram and partnerships with societies or patient organizations might even cost-effectively support patient enrollment.^15^ Considering that most of the accounts of journals, societies and foundations are public, assessing which posts are the most liked or commented on (and what the nature of the comments are, analyzed by thematic analysis) is feasible.

Another research avenue can be to explore whether there is a correspondence between the prominence that journals, societies and medical foundations have in real life vs in the world of Instagram, as done in neurosurgery,^14^ and whether the virtual world is influencing the real one (i.e., regarding conference attendance) or vice versa. Little is known about the time, competence and resources needed to run such accounts. Although these data might be kept private within organizations, looking at cost and benefits would be insightful. Many programs worldwide use Instagram to advertise virtual opportunities. Therefore, one could examine the most popular Instagram accounts that aggregate information for scholarships and bursaries to facilitate neurologists' career progression and what they have in common.

### Professional Networking and Self-Promotion

Instagram can be beneficial for professional networking and self-promotion. Increased social media use among neurosurgeons correlates with greater patient reviews and higher patient scores.^3^ Neurosurgery departments using Instagram accounts showed a significant positive correlation between the total number of reviews and the number of followers (*r* = 0.47), monthly Instagram posts (*r* = 0.46) and videos posted (*r* = 0.56). The quantity of likes received positively correlated with patient ratings (*r* = 0.54).^3^

It remains to be seen whether Instagram can improve publication dissemination and even increase the number of citation-based metrics by the sharing of links to articles, infographics, podcasts and the organization of virtual journal clubs, as already demonstrated using Twitter and Facebook,^16^ and whether a post, a reel or a story (or a combination of them) is the best format to achieve this.

## Patients' Use of Instagram

Patients use Instagram to share their experiences and stories (literary and figuratively) and reach a heterogeneous audience of friends, family, fellow patients, physicians and neurologists. It is the preferred social network for people with multiple sclerosis, who access content about research advances and people's experiences.^16^ Patients, especially women, used Instagram (more than Twitter) to share their personal experiences, as demonstrated in a study on cerebral cavernous malformation.^11^ 84.7% of Instagram posts were made postoperatively and focused on rehabilitation and mobility support.^11^ Before surgery, patients tended to discuss their symptoms, fear of bleeding and mental health.^11^ Hashtags facilitate the retrieval of posts, and an analysis can quickly be performed on who wrote those posts. In the case of epilepsy, for example, the use of just 5 hashtags (#seizure, #seizures, #seizuredisorder, #seizureawareness, #seizurefree) resulted in the collection of 431 posts, 505,368 likes and 420 comments; most of the posts were made by the health and wellness industry (35.0%), survivors (32.7%), organizations, communities, foundations and advocates (15.1%), and doctors (10.2%).^17^

A similar study can be replicated for different neurologic conditions. It would be helpful to analyze whether and how patients “meet” and “connect” in the comments section of Instagram posts, and whether this helped fight stigma or social isolation. It would be enriching to see how celebrities and influencers choose to disclose a neurologic illness through Instagram to put under the spotlight the dynamics and the impact they have on followers (and track how a personal post by a celebrity becomes a de facto public health campaign).^18^

Another idea could be to perform a mixed-method study to analyze who is tagged by patients when they reply to a comment under a post. Yet, the accuracy of posts matters. In the study cited above, 76.8% of the posts contained accurate information (aligned with the guidelines of major health care providers and associations).^17^ The posts written by wellness and health industry professionals, nurses, neuroscience physiotherapists and dieticians had statistically more correct information than those written by patients, survivors and public figures.^17^

## Limitations and Ethical Reflections

Many aspects of using Instagram for educational purposes have limitations and should raise ethical reflections. Misinformation on procedures and medical conditions can fuel a dangerous vortex of misconception because the information is unregulated and unchecked. Instagram hosts promotional/advertising content and the drive to become viral might not always go hand-in-hand with the spread of peer-reviewed medical evidence. The truthfulness of the information uploaded to, and shared through, Instagram is undoubtedly one of the main problems along with the boundaries of its control (*Quis custodiet ipsos custodes?*). People whose interests are not aligned with the promotion of state-of-the-art science and medicine, or patients who do it by mistake, can perpetuate misinformation. Unfortunately, more needs to be done on this. Yet, the advantage of Instagram over other social media, such as TikTok, is that the latter puts “content creation” at the center stage because its more “democratic” algorithm allows everyone to create videos that will potentially go viral, regardless of the number of followers of a specific account. By contrast, it seems that Instagram does not allow this (at least by default), but we still know very little about its algorithm. Instagram has word limits for its users, who, therefore, must be concise, and this might render the description of diseases' nuances more challenging on the patients' side.^11^ Although a key aspect of Instagram is its visual power, being exposed to visual patterns on social media could trigger seizures among people with epilepsy, not to mention addictive behaviors linked to spending too much time on social media platforms.^19^ In light of previous reports showing implicit biases in visual depiction and teaching of medical diseases,^20^ future studies will have to explore the existence of “blindspots” and how implicit biases might influence learning in a visual platform like Instagram.

## Opportunities in Medical Education

Social media has revolutionized how we talk with people, opened a new digital world and broadened the horizons of our interactions. Instagram can contain neurology-related content, give visibility to patients' stories, host the accounts of journals and societies, advertise scholarships, highlight role models, showcase (authentic or humorous) aspects of neurologists' routines and be used for outreach events. Still, the impact of these activities and the evaluation of Instagram as an education tool, specifically for neurology and neuroscience education, are limited. Such a lack of educational research opens many opportunities. Beyond the suggestions for studies included above, Instagram can be a data gold mine because all publicly shared data can be accessed by Instagram users. It is free to use and can be utilized in high-resource and low-resource settings. Several metrics can be collected and analyzed: the number of followers, posts, likes, stories, reels, views and reactions, to mention some of them. These can be used as a basis for educational studies. If correctly added, hashtags cluster topics of interest and make the search quick for learners and educators. Switching from quantitative outcomes to qualitative ones, educators can perform a content analysis of Instagram posts and of the comments below the posts and within the reels. Shedding light on concepts such as interaction, trust and influence has a long history in social sciences, and it can also be beneficial for neurology and neuroscience. Focusing on the emotional side of learning is needed, especially considering that Instagram does not always contribute “to positive or helpful emotions and thoughts” and learners and educators might report feelings of insecurity and self-doubt due to exposure to others.^4^ Another major opportunity lies in the theoretical side of Instagram education. The educational field still has to “elaborate a pedagogical theory”: “technology acceptance theories and learning theories are the most commonly used reference theories, and there is a tendency to rely primarily on technology acceptance models rather than pedagogical models”.^21^ While Instagram was *not* designed for the specific purpose of teaching neurology nor optimized as a tool for medical education research, it can do both. Seen through the Instagram lens, student teaching, patient education and just-in-time learning are not separate categories. The mobile phone screen connects patients with neurologists, students with journals and large organizations with caregivers. When the information is reliable, Instagram can be used as a teaching and learning tool to enrich the educational experience for learners, faculty and the public (Figure).

**Figure:**
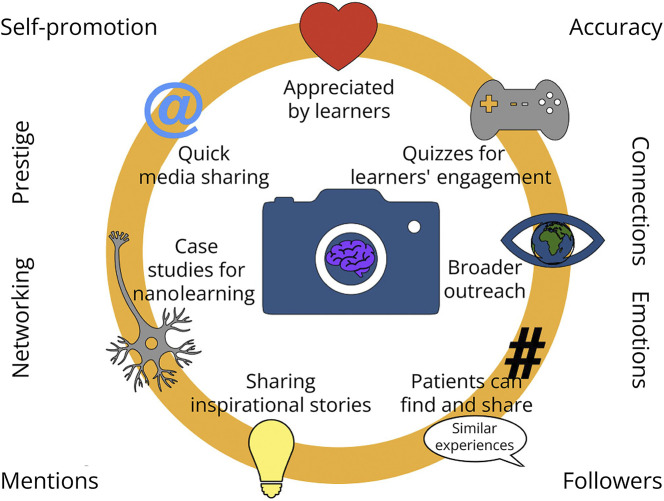
Instagram as an Educational Opportunity in Neurology and Neuroscience Instagram can be used as a teaching and learning tool to enrich the educational experience for learners, faculty and the public.

## Concluding Remarks

Instagram is like a vast sea: its waves cannot be stopped but can be studied, surfed and used for the benefit of the discipline to guide learners and patients and avoid sinking. Exploring the open sea presents dangers and opportunities: minimizing the former and exploiting the latter will make educators, patients and students successful. Instagram as an educational opportunity can no longer be ignored. But now, it is time to refresh our Instagram feed and check who posted what.

## Appendix Author

**Table:**

Name	Location	Contribution
Stefano Sandrone, PhD, MEd	Department of Brain Sciences, Faculty of Medicine, Imperial College London, United Kingdom	Drafting/revision of the manuscript for content, including medical writing for content; major role in the acquisition of data; study concept or design; analysis or interpretation of data

## References

[1] Instagram: statistics & facts. Accessed July 30, 2024. statista.com/topics/1882/instagram/#topicOverview.

[2] Distribution of Instagram users worldwide as of April 2024, by age group. Accessed June 25, 2024. statista.com/statistics/325587/instagram-global-age-group/#:∼:text=As%20of%20April%202024,%20almost,to%2044%20year%20age%20group.

[3] Lamano JB, Riestenberg RA, Haskell-Mendoza AP, Lee D, Sharp MT, Bloch O. Correlation between social media utilization by academic neurosurgery departments and higher online patient ratings. J Neurosurg. 2022;136(6):1760-1772. doi:10.3171/2021.6.JNS2122 34678765

[4] Carpenter JP, Morrison SA, Craft M, Lee M. How and why are educators using Instagram? Teach Teach Educ. 2020;96:103149. doi:10.1016/j.tate.2020.103149 32834464 PMC7380928

[5] Gauthier TP, Bratberg J, Loi K, DiVall MV. Delivery of educational content via Instagram. Med Educ. 2016;50(5):575-576. doi:10.1111/medu.13009 27072461

[6] Yakar F, Jacobs R, Agarwal N. The current usage of Instagram in neurosurgery. Interdiscip Neurosurg. 2020;19:100553. doi:10.1016/j.inat.2019.100553

[7] Graefen B, Fazal N. Revolutionizing education through Instagram in the post-Covid era. Eur J Educ Stud. 2023;10(8). doi:10.46827/ejes.v10i8.4900

[8] Hu J. Microlearning helps Alzheimer's Disease patients. EAI Endorsed Transactions on e-Learning. 2023;9. doi:10.4108/eetel.4321

[9] Purwanto A, Fahmi K, Cahyono Y. The benefits of using social media in the learning process of students in the digital literacy era and the education 4.0 era. J Inform Syst Manag. 2023;2(2):1-7.

[10] Gaini RR, Patel KM, Khan SA, Singh NP, Love MN. A rise in social media utilization by US neurology residency programs in the era of COVID-19. Clin Neurol Neurosurg. 2021;207:106717. doi:10.1016/j.clineuro.2021.106717 34091422 PMC9759490

[11] Gajjar AA, Jain A, Le AH, Salem MM, Jankowitz BT, Burkhardt JK. Cerebral cavernous malformations patient perception analysis via social media. J Neurol Surg A Cent Eur Neurosurg. 2024;85(2):126-131. doi:10.1055/a-1994-9435 36481997

[12] Sandrone S, Carlson C. Gamification and game-based education in neurology and neuroscience: applications, challenges, and opportunities. Brain Disord. 2021;1:100008. doi:10.1016/j.dscb.2021.100008

[13] Saposnik G. Understanding social media: how its popularity could be used to advance medical education in stroke care? J Neurol. 2023;270(8):4096-4102. doi:10.1007/s00415-023-11743-w 37202604

[14] Nouri A, Haemmerli J, Lavé A, et al. Current state of social media utilization in neurosurgery amongst European Association of Neurosurgical Societies (EANS) member countries. Acta Neurochir (Wien). 2022;164(1):15-23. doi:10.1007/s00701-021-04939-4 34313853 PMC8313658

[15] Claus EB, Feliciano J, Benz LS, Calvocoressi L. Social media partnerships with patient organizations for neuro-oncology patient recruitment. Neurooncol Pract. 2020;7(2):143-151. doi:10.1093/nop/npz049 32626583 PMC7318861

[16] Erskine N, Hendricks S. The use of Twitter by medical journals: systematic review of the literature. J Med Internet Res. 2021;23(7):e26378. doi:10.2196/26378 34319238 PMC8367184

[17] Popoola-Samuel HA, Bhuchakra HP, Tango T, Pandya ND, Narayan KL, Pandya N. Instagram and seizure: knowledge, access, and perception of circulating information on the internet. Cureus. 2023;15(7):e41664. doi:10.7759/cureus.41664 37575724 PMC10412441

[18] Myrick JG, Willoughby JF, Francis DB, Noar SM. The impact of celebrity and influencer illness disclosures. Health Commun. 2024:1-4. doi:10.1080/10410236.2024.2326261 38594789

[19] Faelens L, Hoorelbeke K, Cambier R, et al. The relationship between Instagram use and indicators of mental health: a systematic review. Comput Hum Behav Rep. 2021;4:100121. doi:10.1016/j.chbr.2021.100121

[20] Massie JP, Cho DY, Kneib CJ, Sousa JD, Morrison SD, Friedrich JB. A picture of modern medicine: race and visual representation in medical literature. J Natl Med Assoc. 2021;113(1):88-94. doi:10.1016/j.jnma.2020.07.013 32753112

[21] Perez E, Manca S, Fernández-Pascual R, Mc Guckin C. A systematic review of social media as a teaching and learning tool in higher education: a theoretical grounding perspective. Educ Inf Tech. 2023;28(9):11921-11950. doi:10.1007/s10639-023-11647-2

